# A Study of Patients and Nurses’ Perception of the Quality of Pain Management in the Patients Undergoing Surgery in the Departments of Surgery of Rasht Hospitals in 2013

**DOI:** 10.5539/gjhs.v7n7p55

**Published:** 2015-03-26

**Authors:** Tahereh Khalkhali Rad, Shirin Sayad, Maryam Baghaei, Shahla Mola Hossini, Asieh Salahshorian, Mohamad Zare

**Affiliations:** 1 School of Nursing & Midwifery, Islamic Azad University, Tehran Medical Branch, Tehran, Iran; 2 School of Nursing & Midwifery, Shahid Beheshti University, Rasht, Iran; 3 The Faculty Member of Islamic Azad University of Nursing & Midwifery, Tehran Medical Branch, Tehran, Iran; 4 Islamic Azad University of Nursing & Midwifery, Tehran Medical Branch, Tehran, Iran

**Keywords:** perception, pain, surgery, patient satisfaction

## Abstract

**Purpose & Field:**

More than one hundred million people around the world undergo a surgery annually. Although, the surgery itself is a treatment method to relieve pain and discomfort, it can be considered as one of the important factors to make a pain too. Perception and diagnosis of the pain is the most important duty of nurses. Effective pain management after surgery facilitates the patient’s recovery, decreases the length of hospitalization and increases the patient satisfaction. This study aims to investigate the patients and nurses’ perception of the quality of pain management in the patients undergoing an abdominal surgery.

**Methods & Materials:**

The current study is a descriptive research that has been conducted on 204 candidate patients for the abdominal surgery and the nurses who care them in the departments of surgery of Rasht hospitals by using the Simple Random Sampling method. The necessary tools in gathering data for the questionnaire consist of demographic characteristics. Idval, E et al’s Questionnaire for evaluation and pain perception, numerical and visual evaluation tools for the patient and nurse satisfaction with pain relief. Statistical analysis has been made through the 16 version of SPSS software by using descriptive statistics, average and standard deviation.

**Findings:**

The results show that the level of patient satisfaction with providing necessary care to relieve pain was 29.1% (maximum), 20.8% (minimum) and 78.7% to the confidence, environment and all areas, respectively. For the nurses, this level was 32.4% (maximum), 16.4% (minimum) and 77.1% to the performance, environment and all areas, respectively. The maximum level of patient perception of satisfaction with pain relief was 49.1% and for the nurses, it was 37.7% (good level).

**Conclusions:**

The results indicated that the patients’ perception of providing necessary cares to relieve pain and their satisfaction with the pain relief are more than the nurses and in a good level.

## 1. Introduction

Pain is an unpleasant sensory and emotional experience associated with actual or potential tissue damage (Eghbali, 2007) that has been one of the most common facts and events of human life and one of the most important concerns of human from the beginning to now (Tavakoli et al., 2008) and its management is so important in the heath cares that American Pain Society (APS) introduced the phrase “pain as the 5^th^ vital sign” (Eghbali, 2007). According to the annual reports of centers for disease control and prevention, one people out of 4 has suffered from pain for a long term period in the last six months and one people out of 10 suffers from pain that it lasts for one year or more. More than 20% of all medical visits and 10% drug sales are related to the pain (Heidari et al., 2009). Today, the surgery is a solution for many damages and diseases treatment (Hajinezhad et al., 2008). More than one hundred million people around the world undergo a surgery annually (Zakeri Moghaddam, 2011). Although, the surgery itself is a treatment method to relieve pain and discomfort, it can be considered as one of the important factors to make a pain too (Zakeri Moghaddam et al., 2009). Acute post-surgical pain is a type of physiological reaction due to tissue damages and viscus traction or disease (Abed Saeidi, 2011). Statistics show that almost 10%, 20-40% and 40-60% of the patients undergoing surgery suffer from low pain, moderate pain and severe pain, respectively (Zakeri Moghaddam et al., 2011). Pain diagnosis is one of the most important duties of nurses and pain relief is a basis for nursing care (Ghamari Zare, Anousheh, Vanaki, & Hajizadeh, 2008). Almost 85% of the patients hospitalized in departments of surgery suffer from pain (Tavakoli et al., 2008). Effective pain management after surgery facilitates the patient’s recovery and decreases the length of hospitalization (Ghamari Zare et al., 2008). Pain management consists of applying all of the methods in order to prevent and decrease pain (Karampourian & Amini, 2008). A true perception of pain and its management by the patient decreases the anxiety resulted from fear of pain after surgery and the patient can be recovered faster (Zakeri Moghaddam et al., 2009). The difference between patient’s perception and evaluator’s perception leads to a poor pain management (Tavakoli et al., 2008). Another point in the quality of pain management is the patient satisfaction with the pain relief and it is one of the determining standards of performance (Zakeri Moghaddam et al., 2011). Because the patient is an important element of the treatment chain and her/his satisfaction is one of the main purposes of patient care team (Tavakoli et al., 2008). Therefore, with respect to the importance of pain in the patients undergoing surgery and optimum pain management, the current study has been conducted in order to investigate the patients and nurses’ perception of the quality of pain management in two aspects of providing care and patient satisfaction.

## 2. Methods and Materials

This study is a descriptive research that has been conducted from the beginning of November to the second semester of January in 2012 (two and a half months). The study samples selected randomly include 204 patients undergoing abdominal surgery in the first 24 hours after surgery and 204 nurses who work in the departments of surgery of Rasht hospitals.

The necessary tools in gathering data for the questionnaire include three sections as follows:

- Personal information of nurses (8 questions) and patients (7 queations)
- Idval, E et al.’s Questionnaire for evaluation and pain perception of the patients (with 14 questions that consisted of four areas in communication (3 questions), performance (4 questions), confidence (4 questions) and environment (3 questions) and nurses (with 12 questions in the area of confidence, which the environment was omitted)
- Assessment tool of patient satisfaction with pain relief (with the scores range from zero to 10, i.e. zero = dissatisfaction and 10 = complete satisfaction).

In this study, the content validity index was used in order to consider the scientific validity of the questionnaire. Also, the questionnaire was administered to 40 people of the study samples (20 nurses and 20 patients) within 7 days to determine the stability. For the questions about the quality of pain management, Cronbach’s Alpha was calculated 0.82 and 0.87 for the nurses and patients, respectively. The research methodology was that after going to selected hospitals’ departments of surgery (Health Education Centers of Poursina, Razi, Velayat and Private Centers of Aria and Rasool Akram Hospital in the city of Rasht) and providing some necessary explanations, the questionnaires were administrated to the patients undergoing an abdominal surgery (24 hours after surgery) and the nurses who cared them. Then, the questionnaires were gathered after twenty minutes. Sample respondents were asked to complete the items, if they were uncompleted. For the illiterate elderly patients without any attendant, the questionnaires were read and explained by the researcher and the sample respondents’ answers were recorded. Data analysis was carried out using descriptive statistics including the relative frequency distribution tables, average and standard deviation. The data were analyzed by using the 16 version of SPSS software.

## 3. Findings

The study findings suggest that most of the patients were male (56.9%), 29.9% of the patients were between 31-50 years old, 59.8% were hospitalized in the public hospitals, 60.8% were domiciled in the city, 26.5% were the holder of diploma degree from high school, 68.6% were married and 33.8% of them were housewife.

For the nurses who worked in the departments of surgery, the findings suggested that most of them (95.6%) were female, 47.5% of them were between 31-40 years old, 51.4% were married, 96.1% were the holder of BSc degree, 59.8% worked in the public hospitals, 38.2% had an employment contract with their employers, 60.8% had a working experience between 1-9 years and 68.1% worked in shift.

For the nurses’ responses to the questionnaire for providing necessary cares to relieve pain, the maximum response rate of “strongly agree” (51%) was related to the item No. 3 (in area of communication) and the maximum response rate of “strongly disagree” (39.7%) was related to the item No. 7 (in area of performance) (Table 1).

**Table 1:** Relative and absolute frequency distribution of the study samples who work in the departments of surgery in terms of their responses to the questions of “Idval’s questionnaire for the nurses” in 2012

	Item	Questions/Response	Strongly disagree		Disagree		Neither agree nor disagree		Agree		Strongly agree	

			NO.	Percent	NO.	Percent	NO.	Percent	NO.	Percent	NO.	Percent
Communication	1	The necessary information about the types of treatment will be provided before surgery	2	1	10	4.9	14	6.9	102	50	76	37.3
	2	At the beginning of each shift, I have necessary information about the patients pains and the treatment type that they received in the previous shift	0	0	4	2	14	6.9	111	54.4	75	36.8
	3	I cooperate with the physicians to relieve the patient’s pain	0	0	2	1	12	5.9	85	42.2	104	51

Performance	4	After surgery, I will talk to the patients about how to relieve the pain	4	2	0	0	16	7.8	102	50	82	40.2
	5	The personnel support the hospitalized patient to relieve the pain	2	1	8	3.9	12	5.9	94	46.1	88	43.1
	6	I ask the patient about her/his pain when deep breathing in a sitting position or bed turnover	4	2	4	2	20	9.8	128	62.7	48	23.5
	7	At least one time in the morning, noon and night, the patients would be asked to say a number from 0 to 10 to express their pain	81	39.7	18	8.8	69	33.8	16	7.8	20	9.8

Confidence	8	Even without the patient’s request, a painkiller would be used	36	17.6	84	41.2	40	19.6	30	14.7	14	6.9
	9	I have necessary information about the method of pain relief in the patients	2	1	2	1	24	11.8	102	50	74	36.3
	10	I accept the patient’s statements about him/her pain	0	0	2	1	32	15.7	104	51	66	32.4

Environment	11	The necessary environmental conditions would be prepared to keep silence and have a good night’s sleep to the patient	2	1	20	9.8	12	5.9	114	55.9	56	27.5
	12	The patient has a very comfortable and scrumptious room at the hospital	4	2	32	15.7	40	19.6	96	47.1	32	15.7

In the questionnaire for the patients, the maximum answer rate of “strongly agree” (54.4%) was related to the item No. 3 (in area of communication) and the maximum answer rate of “strongly disagree” (40.2%) was related to the item No. 7 (in area of performance) (Table 2).

**Table 2:** Relative and absolute frequency distribution of the study samples after surgery in terms of their responses to the questions of “Idval’s questionnaire for the patients” in 2012

	Item	Questions/Response	Strongly disagree		Disagree		Neither agree nor disagree		Agree		Strongly agree	

			NO.	Percent	NO.	Percent	NO.	Percent	NO.	Percent	NO.	Percent
Communication	1	The necessary information about the types of treatment provided before surgery	5	2.5	10	4.9	25	12.3	77	37.7	87	42.6

	2	At the beginning of each shift, the nurses have necessary information about my pain and the treatment type that I received in the previous shift	0	0	2	1	19	9.3	79	38.7	104	51

	3	The physicians and nurses work together to relieve my pain	0	0	2	1	8	3.9	83	40.7	111	54.4

Performance	4	After surgery, I will talk to the nurses about how to relieve the pain	1	0.5	7	3.4	21	10.3	87	42.6	88	43.1

	5	The personnel support me to relieve the pain	3	1.5	7	3.4	10	4.9	99	48.5	85	41.7

	6	The nurses ask me about my pain when deep breathing in a sitting position or bed turnover	4	2	17	8.3	45	22.5	91	44.6	46	22.5

	7	At least one time in the morning, noon and night, the nurses ask me to express my pain by using the numbers from 0 to 10	82	40.2	32	15.7	55	27	16	7.8	19	9.3

Confidence	8	The nurses give me a painkiller even without my request	16	7.8	98	48	25	12.3	51	25	14	6.9

	9	The nurses help me until my satisfaction with the pain relief	2	1	27	13.2	41	20.1	100	49	34	16.7

	10	The nurses know how to relieve my pain	2	1	3	1.5	23	11.3	78	38.2	98	48

	11	The nurses accept my statements about the pain	4	2	3	1.5	12	5.9	82	40.2	103	50.5

Environment	12	The necessary environmental conditions would be prepared to keep silence and have a good night’s sleep to me	4	2	11	5.4	15	7.4	108	52.9	66	32.4

	13	I have an very comfortable and scrumptious room at the hospital	1	0.5	4	2	50	24.5	80	39.2	69	33.8

	14	There is enough nurses to care the patients	12	5.9	61	29.9	23	11.3	60	29.4	48	23.5

For the four areas of communication, performance, confidence and environment, the performance has the maximum score of 32.4% and the environment has the minimum score of 16.4% among the all scores of nurses’ questionnaire (on a scale of 5, i.e. strongly agree to 1, i.e. strongly disagree). In the questionnaire for the patients, the confidence has the maximum score of 29.1% and the environment has the maximum score of 20.8% (Table 3).

**Table 3:** The level of scores of responses to the questions for “Idval’s Questionnaire in the different areas

Samples	Nurses		Patients	
	
Areas	Score	Percent	Score	percent
Communication	2621	27.7	2655	23.6
Performance	3052	32.4	2974	26.4
Confidence	2216	23.4	3278	29.1
Environment	1546	16.4	2340	20.8
Total	9435	77.1	11247	78.7

For investigating the level of patient satisfaction with the post-surgical pain relief, the nurses and patients were satisfied 37.7 and 49.1%, respectively (Table 4).

**Table 4:** Relative and absolute frequency distribution of the patients and nurses’ perception of the satisfaction with the pain relief in the patients undergoing an abdominal surgery in the departments of surgery of Rasht hospitals

Distribution Satisfaction frequency	Level of satisfaction			

	Nurses		Patients	
	
	Score	Percent	Score	percent
Completely satisfied (8-10)	77	37.7	100	49.1
Intermediate satisfied (6-7)	61	29.9	53	25.9
Poor satisfied (3-5)	43	21.1	42	20.6
Completely dissatisfied (0-2)	23	11.3	9	4.4

## 4. Discussion

For the nurses’ responses to the questions about providing cares to the patients undergoing surgery, the area of communication (question No. 3) had the maximum score of 51% with the response of “strongly agree”.

“From the beginning of the creation of the humans, they have communicated with each other. Communication is to transfer and exchange ideas and opinions between two people or more by using the proper signs and symbols in order to impact and guide each other (),” said Zolfaghari (Moghaddasi, 2010).

Moreover, the maximum score of 39.7% in the area of performance belonged to the question No. 7 with the response of “strongly disagree”. In a study conducted by Heydari, in most cases, the nurses have used the verbal descriptors and the visual chart was not used in any cases. It is probably due to the fact that the nurses have just used their belief as a scale to care the patient and they didn’t use the pain assessment tools, and descriptive pain intensity scales have not yet be used in the departments of surgery practically (Namnabati et al., 2008).

For the nurses’ responses to the questions for providing necessary cares to relieve pain of patients undergoing an abdominal surgery, the most strongly agreed responses were related to the area of communication (48.6%). According to the results obtained from a study conducted by Hajinezhad, the average score for the nurse-patient communication was 4.25 and the standard deviation was 1.14 (the maximum average is 6). Paying attention to the social-psychological aspects of care and especially making an effective communication with patients lead to a higher level of patient satisfaction than the technical aspects of care (Nazari R. et al., 2011). For the area of performance, 44.7% of responses were “strongly agree”. In a study conducted by Ghamari Zare, the quality of nurses’ performance for providing care was in the level of medium (64%) and good (8%) (Yazdi Moghaddam, Memarian, & Mohammadi, 2006). For the area of confidence, the 42.6% of responses was “agree” that in a study conducted by Guninberg, the maximum score was 4.6 that is in accordance with the current study (Idvall, 2007). For the area of environment, the level of agreed responses was 54.3%.

In a study conducted by Nazari, the satisfaction with therapeutic environment was (p = 0.847). Improving the physical space conditions and observing the physical fitness standards is an appropriate base to achieve therapeutic standards, which in turn improve the patients and employees satisfaction (Nazari, 2011).

The method of nurses’ responding to the questions for the level of patient satisfaction with the post-surgical pain relief indicates that most of the patients (37.7%) were strongly agreed. In a study conducted by Moghaddasi, the average of satisfaction with pain was 8.87 (the range of 10 to 0) that is in accordance with the current study (Moghaddasi, 2010).

For the scores of the patients undergoing an abdominal surgery to the questions for providing necessary cares to relieve pain, the question No.7 of the area of performance was given the maximum scores with the “strongly disagreed” responses (40.2%). The question No. 3 of the area of communication was given the maximum scores with the “strongly agreed” responses (54.4%), which the results are in accordance with Guninberg research (Idvall, 2007).

For the patients’ responses to the questions about providing necessary cares to relieve pain, the maximum scores of “strongly agree” were related to the area of performance (56.9%). “A close relationship between the patient and therapeutic staff is needed to relieve pain,” said Eghbali (2007). The maximum response rate of “strongly agree” was related to the area of performance (40%). In the A. Duval research, the score was 3.5 that is not in accordance with this study. The area of confidence had the maximum response rate of “strongly agree” (44.3%). This result is not in accordance with the average of 3.9 in a study conducted by Moghaddasi (2010). For the area of environment, 42.2% of the responses to the questions were “agree”.

Taylor said, “One’s environment and the presence or absence of the people who care him/her can impact on how to experience the pain. Being unfamiliar with the healthcare environment, especially the lamps, noise, lack of sleep and doing a fixed activity can impact on the pain. Failure to comply with environmental conditions can decrease the patient ability to cope the pain.

For the responses to the questions about the patients’ satisfaction with their post-surgical pain relief, most of the sample respondents chose the “strongly satisfied” option (49.1%). According to a study conducted by Yazdi Moghaddam et al. on the post-surgical nursing and recovery care, approximately 49.1% of the patients were dissatisfied when leaving the hospital. The young people were the most satisfied and the elderly patients were the most satisfied persons. According to Tavakoli et al., pain tolerance of women is more than men and the level of women satisfaction is significantly lower than men (p = 0.004).

## 5. Conclusion

Results indicated that the maximum scores (32.4%) and the minimum scores (16.4%) belonged to the areas of performance and environment, respectively. With respect to the scores given by the nurses (9435 scores= 77.1%) compared to the whole scores of questionnaire (12240 scores = 100%), the nurses’ perception of providing the necessary cares to relieve the pain was in a good level. For the patients, the maximum scores (29.1%) and the minimum scores (20.8%) belonged to the areas of confidence and environment, respectively. With respect to the scores given by the sample respondents (11247 scores = 78.7%) compared to the whole scores of questionnaire (14280 scores = 100%), the patients’ perception of providing the necessary cares to relieve pain is in a good level.

37.7% of the nurses and 49.1% of the patients were satisfied with the post-surgical pain relief.
